# Response Surface Methodology-Genetic Algorithm Based Medium Optimization, Purification, and Characterization of Cholesterol Oxidase from ***Streptomyces rimosus***

**DOI:** 10.1038/s41598-018-29241-9

**Published:** 2018-07-19

**Authors:** Akanksha Srivastava, Vineeta Singh, Shafiul Haque, Smriti Pandey, Manisha Mishra, Arshad Jawed, P. K. Shukla, P. K. Singh, C. K. M. Tripathi

**Affiliations:** 1 Microbiology Division, CSIR-Central Drug Research Institute, Lucknow, 226031 India; 2 Academy of Scientific and Innovative Research (AcSIR), Anusandhan Bhawan, New Delhi, 110001 India; 3 Department of Biotechnology, Institute of Engineering & Technology, Lucknow, 226021 Uttar Pradesh India; 4 Research and Scientific Studies Unit, College of Nursing & Allied Health Sciences, Jazan University, Jazan, 45142 Saudi Arabia; 5 Plant Molecular Biology Division, CSIR-National Botanical Research Institute, Lucknow, 226001 India; 6 Department of Biotechnology, Shri Ramswaroop Memorial University, Lucknow, 225003 Uttar Pradesh India

## Abstract

The applicability of the statistical tools coupled with artificial intelligence techniques was tested to optimize the critical medium components for the production of extracellular cholesterol oxidase (COD; an enzyme of commercial interest) from *Streptomyces rimosus* MTCC 10792. The initial medium component screening was performed using Placket-Burman design with yeast extract, dextrose, starch and ammonium carbonate as significant factors. Response surface methodology (RSM) was attempted to develop a statistical model with a significant coefficient of determination (R^2^ = 0.89847), followed by model optimization using Genetic Algorithm (GA). RSM-GA based optimization approach predicted that the combination of yeast extract, dextrose, starch and ammonium carbonate at concentrations 0.99, 0.8, 0.1, and 0.05 g/100 ml respectively, has resulted in 3.6 folds increase in COD production (5.41 U/ml) in comparison with the un-optimized medium (1.5 U/ml). COD was purified 10.34 folds having specific activity of 12.37 U/mg with molecular mass of 54 kDa. The enzyme was stable at pH 7.0 and 40 °C temperature. The apparent Michaelis constant (K_m_) and V_max_ values of COD were 0.043 mM and 2.21 μmol/min/mg, respectively. This is the first communication reporting RSM-GA based medium optimization, purification and characterization of COD by *S. rimosus* isolated from the forest soil of eastern India.

## Introduction

Cholesterol oxidase (COD) is a bi-functional FAD-containing flavo-oxidase enzyme that catalyzes the oxidation of the C3-OH group of cholesterol to produce cholest-5-en-3-one and its isomerization into cholest-4-en-3-one^1,2^. COD has been isolated in the past from various microorganisms such as *Arthrobacter* sp.^3^, *Pseudomonas* sp.^4^, *Brevibacterium sterolicum*^5^, *Mycobacterium* sp.^6^, *Streptomyces violascens*^7^, *Streptomyces cavourensis*^8^, *Streptomyces aegyptia*^9^, etc. COD produced from the microbes is of great commercial interest and widely used for the determination of cholesterol concentration in serum and food samples^10^. COD enzyme has potential applications as a biocatalyst for the bioconversion of sterols and non-steroidal alcohols, exhibits insecticidal properties^11^ as well as involved in bacterial pathogenesis and biosynthesis of macrolide antifungal antibiotics^12^. COD has also been used for the development of electrochemical biosensors^13^ for the study of membrane structures^14^, in the synthesis of polyene macrolide pimaricin antifungal antibiotic in food industry, for the treatment of fungal keratitis^15^, improvement of natamycin production^16^, and the precursor production for hormonal steroids synthesis from cholesterol^17^.

During submerged production of any metabolite, the selection and design of the fermentation medium components and their optimum concentrations are the key factors responsible for the commercial success of the process. The screening and optimization of the fermentation medium plays a crucial role in the cell growth and expression of the desired metabolite affecting the overall productivity. Various non-statistical (conventional) and statistical methods have been practiced widely for the medium optimization. The conventional non-statistical one-factor-at-a-time (OFAT) approach is excessively time-consuming, lacks precision in identifying the critical factors that influence the production of desired metabolite(s), and fails to elucidate the interactions among the factors being studied^18^. To overcome these drawbacks, various statistical methods have efficiently been used for the optimization of the medium composition. Plackett-Burman design (PBD) is the most commonly used technique for the identification of significant process parameters^19^. Central composite design (CCD) is used to study the interaction effect of the factors significantly affecting the product formation. Similarly, Response surface methodology (RSM) is used to describe the behavior of the response in the selected design space^8,20^. Whereas, Genetic algorithm (GA) approach is used for solving both constrained and unconstrained optimization problems based on natural selection. GA is the process that drives the biological evolution process and can be used to optimize its operational space representing process variables with a view to maximize the process yields^21^.

In the present study, attempts have been made by employing RSM coupled GA approach to quantitatively evaluate the individual and combined interaction effect(s) of the medium components on COD production from *Streptomyces rimosus* isolated from the eastern part (Chhatisgarh) of India, along with the purification and characterization of the said enzyme.

## Results

### Medium optimization using classical OFAT method

**Figure 1:**
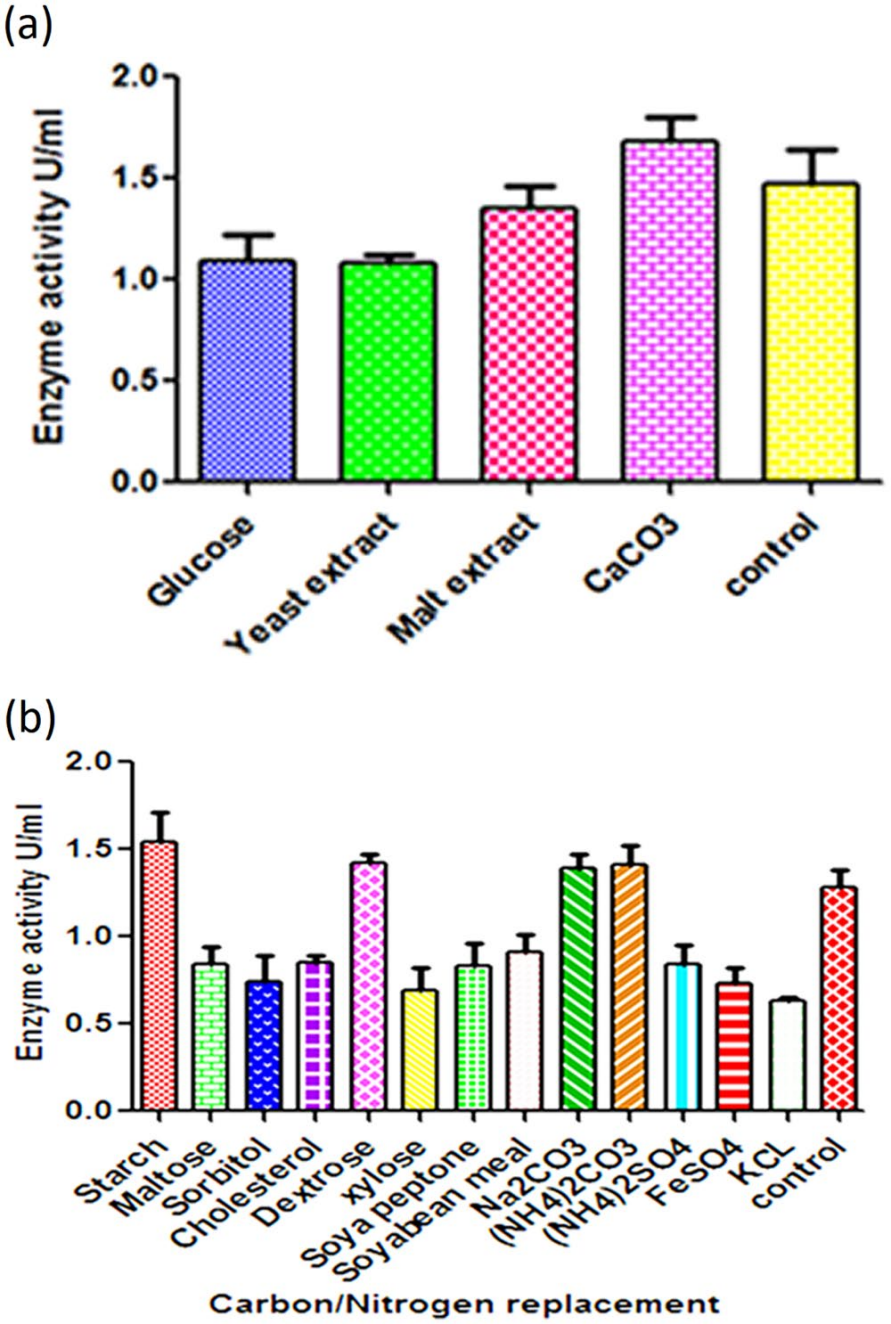
(**a**) Effect of removed components from medium on enzyme activity. (**b**) Effect of carbon/nitrogen replacement on COD production.

Cholesterol oxidase indicator agar method is one of the most promising method for the confirmation of COD production from the microbial strains. The isolate, *S. rimosus* MTCC 10792 was capable of changing the color of the medium into intense brown. This color change confirmed the conversion of cholesterol into 4-cholesten-3-one and H_2_O_2_ to form azo-compound (Schiff’s base). Phylogentic, physiological and biochemical characterization of the microbal strain *S. rimosus* MTCC 10792 has been published earlier^22^. OFAT method was successfully used to investigate the effects of various medium components including carbon and nitrogen production. The elimination of glucose and yeast extract from the production medium had a significant effect on COD production and it decreased the productivity ~20 and 30%, respectively, in comparison to the control. Hence, these components were considered essential for COD production (Fig. 1a). For the replacement experiments, carbon and nitrogen sources were selected based on the supplementation experiment (data not shown). Among the carbon sources, dextrose and starch were found to be effective in enhancing the production of COD by ~20 and 28%, respectively, in comparison with the control (Fig. 1b).

### Medium optimization using RSM coupled GA approach

PBD was used to screen and investigate the actual variables (medium components) responsible for significantly affecting the production of COD. The results of PBD revealed that yeast extract (t = 16.47695; p = 0.00001), dextrose (t = 4.963093; p = 0.000284), starch (t = 0–3.65139; p = 0.00222) and ammonium carbonate (t = 1.395358; p = 0.09655) had high t- (test statistics) and low p-values. High t- and a low p-value of these medium components showed their potential impact on COD yield (Table 1). The above mentioned components were further used in CCD to study their interaction effect(s). The observed and the predicted values of COD enzyme activities have been given in Table 2. The regression coefficient of each variable in terms of linear, quadratic and interaction, along with p-values at ≥90% confidence shown in Table 3. The linear effect of yeast extract (p = 0.000), dextrose (p = 0.0254) and starch (p = 0.000), quadratic effect of yeast extract (p = 0.0007) and ammonium carbonate (p = 0.0075), and interaction effect of starch with yeast extract (p = 0.000), dextrose with starch (p = 0.000) and dextrose with ammonium carbonate (p = 0.0250) were found significant. To validate the regression coefficient, analysis of variance (ANOVA) was performed, which suggested the adequacy of the second-order response surface model (Table 4). The Fisher’s F-test having low probability value (p < 0.05) signified high statistical significance of the regression model. High correlation coefficient (R) value denotes an excellent correlation among the independent variables^23^, and in the present study, the medium components were explained by the high value of correlation coefficient (R = 94%). The goodness of fit of the model was explained by the determination coefficient (R^2^) and its value was lying between 0 to1 (as the value of R^2^ is closer to 1, better is the estimation of regression equation). In general, the coefficient of determination reflects the percentage variation explained by the regression equation. In our experiments, the value of R^2^ (0.89) suggested that the generated second order polynomial model could explain the 89% of the total data. RSM was applied using MATLAB7.7 software program and yielded following second-order polynomial equation:

1 $$\begin{array}{c}{\rm{Y}}=0.1801+{2.8746}^{\ast }{\rm{x}}(1)+{0.4696}^{\ast }{\rm{x}}(2)+{1.3511}^{\ast }{\rm{x}}(3)-{32.558}^{\ast }{\rm{x}}(4)\\ \,\,\,\,-\,{0.0125}^{\ast }{\rm{x}}{(1)}^{\ast }{\rm{x}}(2)-{1.9594}^{\ast }{\rm{x}}{(1)}^{\ast }{\rm{x}}(3)+{48.375}^{\ast }{\rm{x}}{(1)}^{\ast }{\rm{x}}(4)\\ \,\,\,\,-\,{1.175}^{\ast }{\rm{x}}{(2)}^{\ast }{\rm{x}}(3)+{65.5}^{\ast }{\rm{x}}{(2)}^{\ast }{\rm{x}}(4)+{16.0625}^{\ast }{\rm{x}}{(3)}^{\ast }{\rm{x}}(4)\\ \,\,\,\,+\,{0.4880}^{\ast }{\rm{x}}{(1)}_{.}^{2}-{0.112}^{\ast }{\rm{x}}{(2)}_{.}^{2}+{0.0142}^{\ast }{\rm{x}}{(3)}_{.}^{2}-{302.3011}^{\ast }{\rm{x}}{(4)}_{.}^{2}\end{array}$$

where, Y = Product Yield, x(1) = Yeast Extract, x(2) = Dextrose, x(3) = Starch, x(4) = Ammonium carbonate.

**Table 1:** Plackett-Burman design and effect of medium components on COD production.

Run	Independent variables							E A U/ml
	A	B	C	D	E	F	G	
1	0.1	0.25	0.1	0.05	0.25	0.0025	0.0025	1.64
2	0.1	0.25	0.1	0.05	0.25	0.0075	0.0075	1.631
3	0.1	0.25	0.3	0.15	0.75	0.0025	0.0025	1.561
4	0.1	0.75	0.1	0.15	0.75	0.0025	0.0075	1.512
5	0.1	0.75	0.3	0.05	0.75	0.0075	0.0025	1.801
6	0.1	0.75	0.3	0.15	0.25	0.0075	0.0075	1.825
7	0.3	0.25	0.3	0.15	0.25	0.0025	0.0075	2.423
8	0.3	0.25	0.3	0.05	0.75	0.0075	0.0075	2.339
9	0.3	0.25	0.1	0.15	0.75	0.0075	0.0025	2.168
10	0.3	0.75	0.3	0.05	0.25	0.0025	0.0025	2.506
11	0.3	0.75	0.1	0.15	0.25	0.0075	0.0025	2.277
12	0.3	0.75	0.1	0.05	0.75	0.0025	0.0075	2.075
Effect	0.6368	0.0393	0.1918	−0.0377	−0.1411	0.0539	−0.0248	
V_eff_	0.001494							
S.E.	0.038652							
t- value	16.4770	1.0185	4.9631	−0.9762	−3.6513	1.3954	–0.6416	
p-value	0.00001	0.166247	0.000284	0.17599	0.00222	0.09655	0.26777	

*Note*: A (yeast extract) B (malt extract), C (dextrose), D (calcium carbonate), E (starch), F (ammonium carbonate), G (sodium carbonate) are the independent variables. All the variables were measured in g/100 ml. The p-values less than 0.05 are significant.

**Table 2:** CCD with observed and predicted values of CO activity.

Yeast Extract	Dextrose	Starch	Ammonium carbonate	Enzyme activity U/ml		
				Observed	Predicted	Residual
0.4	0.4	0.8	0.02	1.694	1.698545	−0.004545
0.4	0.4	0.8	0.02	1.7	1.698545	0.001455
0.6	0.6	0.4	0.01	2.168	2.164430	0.003570
0	0.4	0.8	0.02	0.66	0.788276	−0.128276
0.2	0.2	0.4	0.01	0.76	0.749693	0.010307
0.4	0.4	1.6	0.02	1.96	2.097274	−0.137274
0.4	0.4	0.8	0	1.25	1.326244	−0.076244
0.8	0.4	0.8	0.02	2.788	2.823809	−0.035809
0.2	0.2	1.2	0.01	1.534	1.394519	0.139481
0.4	0.4	0.8	0.02	1.7	1.698545	0.001455
0.4	0	0.8	0.02	1.33	1.402118	−0.072118
0.4	0.4	0.8	0.04	1.8	1.854525	−0.054525
0.6	0.2	1.2	0.03	2.427	2.344019	0.082981
0.6	0.2	0.4	0.03	2.023	2.019173	0.003827
0.4	0.8	0.8	0.02	1.926	1.977144	−0.051144
0.4	0.4	0.8	0.02	1.69	1.698545	−0.008545
0.4	0.4	0.8	0.02	1.7	1.698545	0.001455
0.4	0.4	0	0.02	1.35	1.283244	0.066756
0.4	0.4	0.8	0.02	1.71	1.698545	0.011455
0.4	0.4	0.8	0.02	1.7	1.698545	0.001455
0.4	0.4	0.8	0.02	1.69	1.698545	−0.008545
0.2	0.6	1.2	0.03	1.855	1.743433	0.111567
0.2	0.6	0.4	0.03	1.2	1.193526	0.006474
0.6	0.6	1.2	0.01	1.939	1.832878	0.106122
0.4	0.4	0.8	0.02	1.71	1.698545	0.011455
0.4	0.4	0.8	0.02	1.7	1.698545	0.001455
0.4	0.4	0.8	0.02	1.69	1.698545	−0.008545

*Note:* The concentration units of yeast extract, dextrose, starch and ammonium carbonate were in g/100 ml.

**Table 3:** Estimated regression coefficients for COD yield.

Effect	Coefficients	SE	Wald Stat.	p-value
Intercept	−0.1801	0.26183	33.64008	<0.0001
Var1	2.8746	0.50285	49.49338	<0.0001
Var1^2^	0.4879	0.24010	11.39588	0.000736
Var2	0.46960	0.43527	4.99551	0.025413
Var2^2^	−0.112	0.17871	0.48697	0.485280
Var3	1.35105	0.21073	39.91996	<0.0001
Var3^2^	0.01418	0.04532	1.43576	0.230827
Var4	−32.5579	8.95763	0.00056	0.981069
Var4^2^	−302.301	74.69575	7.13023	0.007579
Var1*Var2	−0.01250	0.53368	1.29396	0.255319
Var1*Var3	−1.95938	0.18269	75.60701	<0.0001
Var2*Var3	−1.1750	0.19853	19.22840	0.000012
Var1*Var4	48.37499	10.43085	1.10629	0.292891
Var2*Var4	65.4999	11.06510	5.01864	0.025076
Var3*Var4	16.0625	3.96512	0.33786	0.561066

*Note*: Var1 = yeast extract, Var2 = dextrose, Var3 = starch, Var4 = ammonium carbonate. SE = standard error. Wald Stat = Wald statistics. The p-values less than 0.05 are significant.

**Table 4:** Analysis of variance (ANOVA) of the model derived from response surface methodology.

Effect	Analysis of Variance; DV: Var5				
	Sums of Squares	df	Mean Squares	F	p-value
Regress	4.445999	4	1.111500	48.67242	<0.0001
Residual	0.502399	22	0.022836		
Total	4.948399				

**Figure 2:**
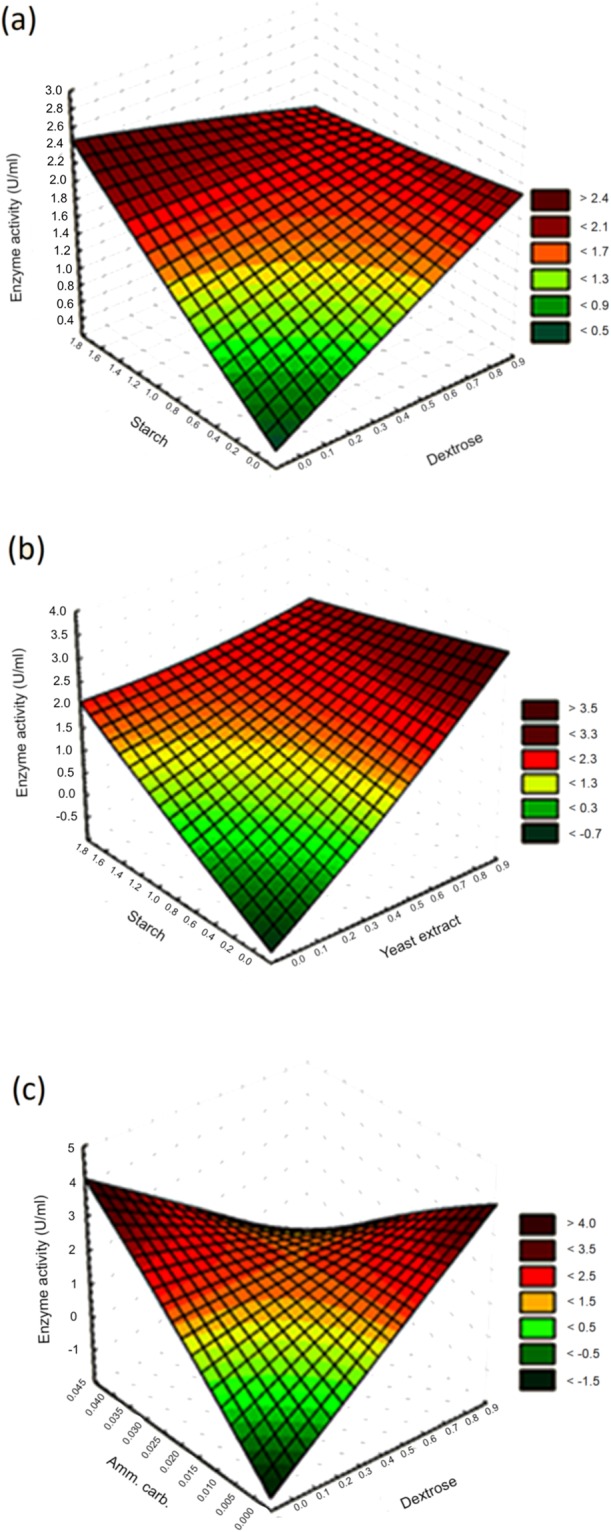
(**a**) 3D surface plot of dextrose and starch. (**b**) 3D surface plot of yeast extract and starch. (**c**) 3D surface plot of dextrose and ammonium carbonate.

The response surface plot(s) was generated using two independent variables to visualize COD yield, while the other variables were kept constant at their center value. The relationship between COD yield and the variables (medium components) have been shown as a curved response surface within the design space^24^. The three-dimensional (3D) response surface plots were obtained using STATISTICA 12.0 (Fig. 2a–c) software program. It is evident from the surface plot (Fig. 2a) of yeast extract and starch that a high concentration of yeast extract and a low to medium concentration of starch were necessary for high COD production. Likewise, it was also observed by analyzing other interaction surface plots that central values of dextrose and starch (Fig. 2b) with high values of ammonium carbonate (Fig. 2c) were crucial for the maximum COD production.

The regression model (Eq. 1) generated through RSM was analyzed by genetic algorithm (GA) approach and executed. The production of COD was significantly increased up to 5.41 U/ml in comparison to the COD production (1.5 U/ml) using an un-optimized medium. Using the defined criteria, the response of the model successfully reached to its optimum value after eleven generations [Supplementary Figure S1(a,b)]. The algorithm found a maximum production of the enzyme in the given experimental boundaries at the optimized values of the variables. The optimum concentration of the four variables was found to be 0.99 g/100 ml yeast extract, 0.8 g/100 ml dextrose, 0.1 g/100 ml starch and 0.05 g/100 ml ammonium carbonate, and the observed COD production after the medium optimization process was 5.25 U/ml, which was very close to the predicted COD production of 5.41 U/ml. The experimentally verified COD yield increased significantly by ~3.6 folds [i.e., from 1.5 U/ml (using un-optimized) to 5.25 U/ml (using RSM-GA optimized medium).

### Purification of COD

The culture filtrate precipitated with ammonium sulphate (70% saturation) was purified sequentially on ion-exchange followed by gel-permeation chromatography at 4 °C. The purification steps have been illustrated in Table 5. The precipitated enzyme solution was loaded on to a DEAE-cellulose column and elution was performed with the gradient of NaCl (10–50 mM) in 100 mM potassium phosphate buffer (pH 7.0) [Supplementary Figure S2(a)]. The fractions showing maximum COD activity were pooled and further purified by gel filtration chromatography with Sephadex G-100 column. The elution was done with 30 mM NaCl in 100 mM potassium phosphate buffer (pH 7.0). The elution profile and SDS are given in Supplementary Figure S2(a,b), respectively. The purified enzyme preparation gave a single band on SDS-PAGE with the estimated molecular mass of 54 kDa [(Supplementary Figure S2(b)]. The extracellular COD was purified 10.31 folds with specific activity 12.37 U/mg using ion-exchange and gel-filtration chromatography. The purified enzyme was analyzed using Mascot mass fingerprinting database on MALDI -TOF for protein similarity search. MS/MS data of the protein was matched with COD of *Streptomyces* sp. on the basis of four peptides (a total of 90 amino acids) with 16% coverage and a Mascot score of 265 [Supplementary Figure S2(c)].

**Table 5:** Purification of COD from *Streptomyces rimosus*.

Purification step	Protein (mg)	Activity (U)	Specific activity (U mg^−1^)	Yield (%)	Purification (fold)
Culture supernatant	1550	1865	1.20	100	1
Ammonium sulphate	550	1470	2.67	78.82	2.22
DEAE cellulose	220	1150	5.22	61.62	4.35
Sephadex G-100	80	990	12.37	53.08	10.31

### Characterization of purified COD

**Figure 3:**
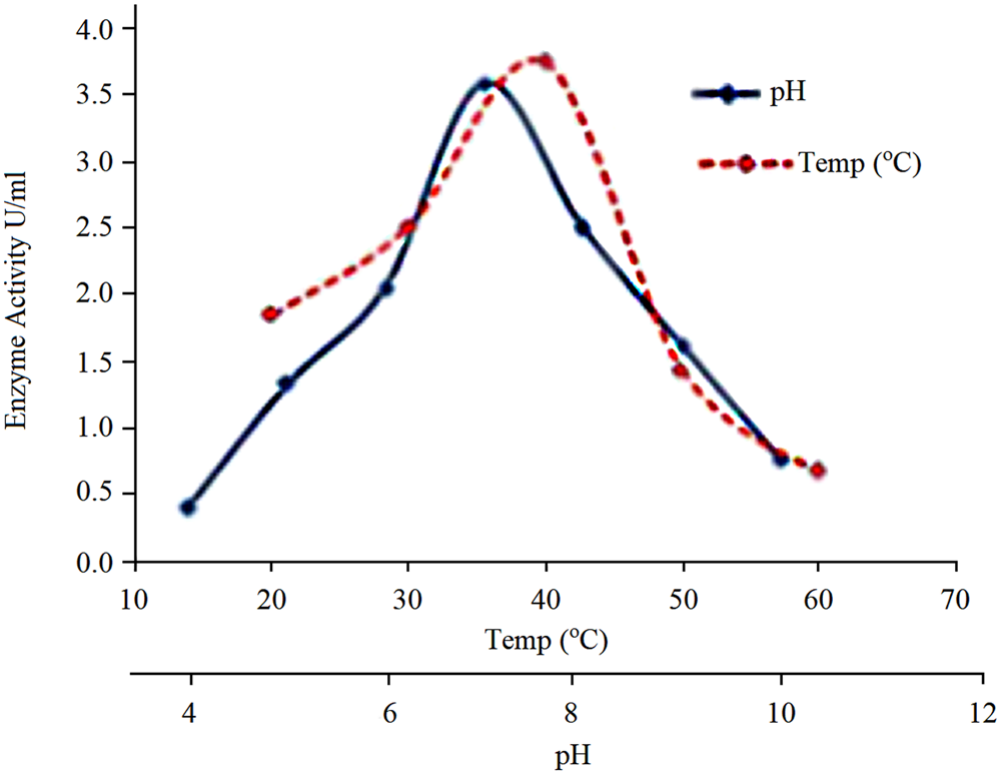
Effect of pH and temperature on purified cholesterol oxidase.

The purified COD was most active at pH 7.0 and retained 70–80% of its initial activity at pH 6.0–8.0; and any change in the pH resulted decrease in its activity (Fig. 3). The effect of the temperature on COD activity was checked between 20–60 °C and the highest activity was observed at 40 °C that decreased upto 70% at higher temperatures (60 °C).

**Figure 4:**
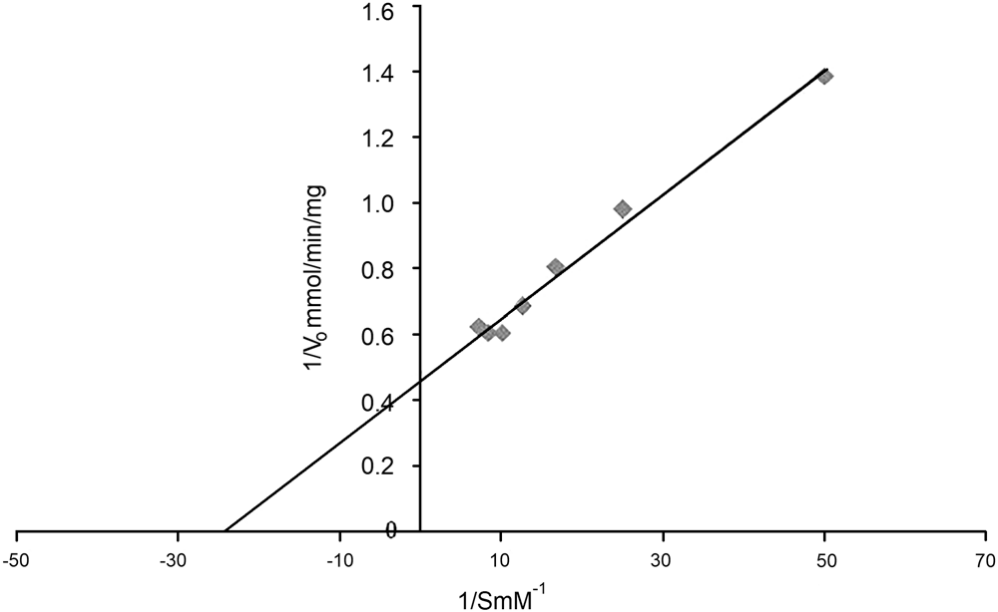
Lineweaver-Burk plot for COD using cholesterol as substrate.

Metal ions, Ca^2+^, Mg^2+^, Ni^2+^ at 5 mM concentration did not show any effect on COD activity. However, Ag^2+^ and Hg^2+^ inhibited the activity to 55 and 50%, respectively (Table 6). The effect of solvents and detergents was observed on the enzyme activity that showed 100% relative activity in the presence of sodium cholate and an enhancement 80% in the presence of Triton X-100, while denaturing agents like SDS and DTT decreased the relative activity by 20 and 30%, respectively. Although, COD activity was not much (~10–30%) affected by all the examined solvents except chloroform, yet only ethanol and isopropanol (10% v/v) showed enhancement in COD activity by 60–81% (Table 7). Among different substrates of COD, β-cholestanol and β-sitosterol were rapidly oxidized, whereas epiandrosterone; ergosterol with 15 and 12% relative activity, respectively, were oxidized slowly (Table 8). According to Lineweaver-Burk plot method, the K_m_ value for cholesterol was determined as 0.043 mM and V_max_ as 2.21 μmol/min/mg (Fig. 4). The identification of the prosthetic group in the purified COD (1 mg/ml) was observed in the UV visible absorption spectrum, which revealed dual absorption maxima at 280 and 380 nm (Supplementary Figure S3).

**Table 6:** Effects of metal ion on COD activity.

Metal ion (5 mM)	Relative activity (%)
Control	100
Ca^+2^	98
Cu^+2^	96
Mg^+2^	100
Ni^+2^	99
Fe^+2^	98
Pb^+2^	65
Ag^+2^	55
Hg^+2^	50
Zn^+2^	65
Mn^+2^	99

**Table 7:** Stability of COD in organic solvents and detergents.

Solvents (10%)	Relative activity (%)
Control	100
DMSO	89
Methanol	110
Ethanol	170
Ethyl acetate	105
Acetone	65
Isopropanol	180
Chloroform	20
Benzene	80
**Detergents (0.5%)**	**Relative activity (%)**
Control	100
Tween 80	120
Triton X-100	180
Triton X-100(1%)	80
Triton X-114	115
Sodium cholate	100
SDS	20
Beta-mercaptoethanol	60
DTT	30

**Table 8:** Substrate specificity of COD.

Substrate (0.5 mM)	Relative activity (%)
Cholesterol	100
β-Cholestanol	96
β-Sitosterol	88
β-Stigmasterol	68
Pregnenolone	46
Dehydroepiandrosterone	30
Epiandrosterone	15
Ergosterol	12

## Discussion

COD is a FAD-containing enzyme having potential applications in medical and enzyme industries^12^. Various microbes, for e.g., *Pseudomonas* sp., *Brevibacterium sterolicum*, *Streptoverticillium cholesterolicum* and some species of *Streptomyces* like *S. violascens*, *S. parvus*, etc. are known to produce extracellular COD^25,26^. Singh and Rai (2012) reported the isolation of an extracellular COD producing strain from the forest soil of Chhattisgarh, India^22^.

The production of COD was confirmed using screening experiments in which COD converted cholesterol into 4-cholesten-3-one and H_2_O_2._ This hydrogen peroxide form azo-compound with Schiff’s base present in the medium and changed the color of the medium into dark brown^27^. The isolated strain characterized as *S. rimosus* produced 1.5 U/ml of COD by using un-optimized medium. In the present study, attempts have been made to enhance the COD production from *S. rimosus* by applying various statistical/mathematical optimization techniques. The results obtained from the classical experiments suggested that the presence of yeast extract, starch or glucose in the production medium were responsible for the enhancement of COD production. These results were further verified by using statistical Plackett-Burman Design. Earlier, Yazdi *et al*.^28^ have reported that starch and glucose are the promising carbon sources for COD production by *S. fradiae*.

The microbes can preferentially utilize glucose via catabolite repression and convert it into ribose^29,30^ that may be further utilized in the synthesis of FAD, which in turn increase the concentration of COD. Here in this study, none of the organic nitrogen sources except yeast extract were found to be supportive for the production of COD. From the previous report, it has been well established that yeast extract contains a variety of essential amino acids and growth factors capable of promoting cell growth, metabolism and COD production^31^. Kreit *et al*.^32^ have reported the production of membrane-bound COD from *Rhodococcus* sp. and found that inclusion of 0.3% (w/v) yeast extract in the medium resulted in maximum COD production. Similarly, Lee *et al*.^33^ have reported that yeast extract was found to be the best nitrogen source (0.4–0.5% w/v) for COD production from *Rhodococcus equi*. A higher concentration of starch results in higher viscosity that hampers oxygen transfer and reducing its availability for the microorganism. Higher availability of dextrose and scarcity of oxygen reduces ATP yield, produces acidic by-products and affects the growth and protein expression, which is commonly known as Crabtree effect^34^.

The effect of various inorganic nitrogen sources on COD production was also investigated. Ammonium carbonate increased the enzyme production by 30%. This finding is supported by the previous report, where ammonium salts have been reported to show maximum influence on COD production by *Arthrobacter simplex*^35^.

The production of COD from *Streptomyces* varies from strain to strain, for e.g., 2.45, 3.14, and 2.21 U/ml from *S. badius, S. fradiae*, and *S. lavendulae*, respectively, as stated in different reports. Also, various statistical optimization techniques have been used successfully to increase the COD production in the past by using different *Streptomyces* strains. Chauhan *et al*.^31^ reported 2.48 folds (2.21U/ml) increase in overall COD production from *S. lavendulae* using an orthogonal array and RSM approach. Similarly, Lee *et al*.^18^ employed RSM and observed four folds (0.242 U/ml) increase in COD productivity using *Rhodococcus equi*. Whereas, in the present study, high COD production (5.41 U/ml) was achieved using *S. rimosus*, which reflects 3.6 folds increased COD productivity by applying RSM coupled GA approach. To the best of our knowledge this is first communication reporting high COD production by using RSM coupled GA approach from *S. rimosus* isolated from the forest soil collected from an eastern part of India. Microbial CODs generally have neutral pH optima and possess stability over a wide range. The enzymes have temperature optima in the range of 40–60 °C. The optimum temperature (70 °C) of COD from *S. fradiae* is the highest among the enzymes reported so far^36^. The addition of Ca^2+^, Mg^2+^, Ni^2+^ did not show any effect on COD activity. However, Ag^2+^ and Hg^2+^ reduced the enzyme activity by 55 and 50%, respectively. In many cases, COD activity is markedly inhibited by -SH inhibitor (Hg^2+^ or Ag^2+^)^37^. Earlier communications reported the inhibition of the enzyme activity in the presence of Hg^2+^ and Ag^2+^ from *S. violascens* and *Streptomyces* sp. SA-COO, respectively^7,38^. The effect of solvents and detergent was studied on the enzyme activity. COD activity increase by 80% in 0.5% v/v solution of Triton X-100, whereas denaturing agent like SDS and DTT reduced the residual COD activity by 20 and 30%, respectively in comparison with the control. Reverse micellar system formed by the combination of enzymes and surfactants/organic solvents; these interactions may cause complete/partial denaturation of the enzyme or may enhance or diminish its catalytic potential^39^. At low concentration of propan-2-ol, there is no formation of mixed micellar structures incorporating COD from *Streptomyces hygroscopicus* and *Brevibacterium sterolicum*, with cholesterol^40^. In the present study, COD activity from *Streptomyces rimosus* (Table 7) showed 70–80% increment in (10%) ethanol and isopropanol. Pollegioni *et al*.^41^ reported that COD activity of *B. sterolicum* is rapidly decreased retaining only 20% activity; whereas, COD from *S. hygroscopicus* retained 70% of the initial activity in the presence of 30% propan-2-ol as compared to the control. Doukyu *et al*.^37^ reported the stability of COD in the presence of 0.5% detergents at various temperatures together with commercially available enzymes of *Streptomyces* sp., *Cellulomonas* sp., *Nocardia* sp., *N. erythropolis* and *Pseudomonas* sp. Since cholesterol is an insoluble compound, detergents are often added to the reaction mixture to act as a solubilizing agent^42^. Isobe *et al*.^43^ reported a detergent-tolerant COD from *γ-Proteobacterium* Y-134, which retained more than 80% of its original activity in 0.5% Triton X-100 and sodium cholate after incubation for 1 h at 60 °C. In the present study, β-cholestanol was oxidized at a higher rate. It was also supported by the study of Kamei *et al*.^44^, where they reported higher substrate specificity for β-cholestanol by *S. violascens*. The enzymes from *Chromobacterium* sp. DS-1^37^, *Streptomyces* sp. SA-COO^38^ and *S. violascens*^44^, oxidized pregnenolone at a high rate.

The Michaelis constant K_m_ is the substrate concentration at which the reaction rate is half of V_max_. Our study showed K_m_ value of 0.043 mM and V_max_ as 2.21 μmol/min/mg, which were much higher than earlier reports obtained from other microbial sources, like *Nocardia* sp. [K_m_ value was 69 mM]^45^, *Brevibacterium sterolicum* [1.1 mM]^46^, and *Streptomyces* sp. [101.3 μM and 21.93 μM/min/mg]^10^.

From the production and purification point of view, the processes for extracellular enzymes must be relatively simpler and commercially viable. High yield of extracellular COD, having tolerance for detergents and organic solvents, with a low-cost medium are the advantageous features of the process being reported here. Further, simple and economical enzyme purification steps based on ion-exchange and gel filtration chromatography are suitable for the analytical applications and other research purposes. Also, this study paves the way for further utilization of *S. rimosus* MTCC 10792 strain for metabolic engineering applications by constructing recombinant strains and for other industrial and biotechnological applications.

## Conclusion

We conclude that RSM-GA amalgamated approach is an efficient and successful strategy for the statistical optimization of the medium components for extracellular COD production. Under optimized medium conditions, 3.6 folds higher COD production was observed as compared to the control (un-optimized) medium. Overall, based on the present findings, we can affirm that *S. rimosus* MTCC 10792 can be used as a potent microbial strain to produce COD at different scales and hybrid approaches (statistical/mathematical) for media optimization greatly help in increasing the COD production and paves the way for a commercial-scale process for COD production and its purification. This study also warrants for further investigations pertaining to in-depth phylogenetic, structural as well as metabolic characterization of the bacteria for future experimental studies and applications.

## Materials and Methods

### Microbial strain, profiling, and growth conditions

The microbial strain used in the present study was isolated from the soil sample collected from the preserved forest ecosystem (21°30′N, 82°0′E) of Chhattisgarh, India. The strain was deposited at Microbial Type Culture Collection (MTCC), Institute of Microbial Technology (IMTECH),Chandigarh, Punjab, India as *Streptomyces rimosus* MTCC 10792^22^. The 16S rRNA sequence of the strain has been submitted to the NCBI GenBank database under the accession number JF915305^22^. Submerged fermentation was performed in yeast and malt extract with glucose (YMG) medium using yeast extract 4.0 g/l, malt extract 10.0 g/l, dextrose 4.0 g/l, and calcium carbonate 2.0 g/l. The initial pH of the prepared medium was maintained as 7.0 and the bacterial culture was incubated in triplicate on a rotary incubator shaker (180 rpm) at 30 °C for 96 h. The culture broth was centrifuged at 11086 g for 15 min at 4 °C and the supernatant was used for the enzyme assay.

### Enzyme assay

The enzymatic activity assay of COD is based on the conversion of cholesterol to 4-cholestene-3-one. The assay was performed by following the method of Allain *et al*.^47^. In this assay, hydrogen peroxide couples with 4-aminoantipyrine and phenol by peroxide to produce quinoneimine dye showing maximum absorption at 500 nm. The reaction buffer was prepared by adding 19.8 mM phenol and 1.5 mM 4-aminoantipyrine in 94 mM potassium phosphate buffer (pH 7.0). A reaction mixture (3.03 ml) was prepared by adding 2.8 ml of above stated phenol, 0.2 ml of 0.9 mM cholesterol (dissolved in 1 ml of 0.35% Triton X-100), 0.01 ml of 19 units of peroxidase from horseradish and 0.02 ml of the enzyme solution. The reaction was performed at 37 °C for 10 min and terminated by heating at 100 °C for 5 min. The reaction mixture was cooled at room temperature (25 °C) and the rate of reaction of product (4-cholesten-3-one) formation was determined by measuring the color developed at 500 nm. One unit of COD enzyme has been defined as the quantity of the enzyme required to produce 1.0 μmol of 4-cholesten-3-one per min at pH 7.0 and at 37 °C. The concentration of the protein/enzyme was measured by the method of Lowry *et al*.^48^, using bovine serum albumin (BSA) as a standard.

### Screening of the medium components by using classical OFAT experiments

Yeast and malt extract with glucose (YMG) medium was screened for its components for the production of COD. To increase the production of COD enzyme, different optimization experiments were carried out using the classical one-factor-at-a-time (OFAT) method, which involved varying one parameter at a time and keeping the others constant at pre-defined levels. The classical method was used to investigate the effects of medium components, like, carbon and nitrogen on COD production. The effects of medium components on COD production was observed through sequential removal experiments, where, the medium components were removed from the culture medium one-by-one and their effects were observed on COD production after 96 h of bacterial growth. Carbon and nitrogen supplements showing improved results in the supplementation experiments (data not shown) were selected as the sole carbon and nitrogen sources, and their effects on COD production were examined by the replacement experiment. Dextrose was replaced with maltose, starch, and sorbitol. Similarly, yeast extract was replaced with soybean meal and soya peptone at the concentration of 1% in the production medium.

### Statistical medium optimization

Plackett-Burman design (PBD) was used to determine the major factors affecting the production of COD by evaluating the seven medium components, *viz*. yeast extract, malt extract, dextrose, CaCO_3,_ starch, (NH_4_)_2_CO_3_ and Na_2_CO_3_, in twelve trials. For each variable, two levels i.e., high (H) and low (L), were considered. The effect of each variable was examined by the following equation:

2 $${E}_{(X1)}=\frac{\sum C{1}_{H}-\sum C{1}_{L}}{N}$$

Where, *E*_*(X1)*_ is the concentration effect of the tested variable. In the Eq. 2, *C1*_*H*_ depicts the COD activity from the trials corresponding to the high (H) concentration of the factors, *C1*_*L*_ represents are the COD activity from the trials corresponding to the low (L) concentration of the factors, and N is the total number of the trials. The variance of the dummy variable was calculated for the estimation of the experimental error by the given equation.

3 $${V}_{eff}=\frac{\sum {({E}_{d})}^{2}}{n}$$

Where, *V*_*eff*_ is the variance in the concentration effect, *n* is the number of dummy variables, and *E*_*d*_ is the concentration effect for the dummy variables^19^.

The standard error (*S.E*.) was described by the square root of the variance of the effect, i.e.

4 $$S.\,E.=\sqrt{{V}_{eff}}$$

The significance level of each concentration effect was determined by using the student’s *t*-test^49^,

5 $${t}_{(X1)}={E}_{(X1)}/S.\,E.$$

The variables with the confidence levels greater than 90% were considered for significantly influencing the COD productivity. The most significant variables obtained through PBD were optimized using RSM and GA approaches. The experiments were performed according to four-factor CCD and the concentration of each variable was varied at five experimental levels (−2, −1, 0, +1, +2) containing eight-star points. Ten replicas of the center-points were employed to achieve the second order polynomial model in a total of twenty-seven experiments. All experiments were performed in triplicate and the mean values were used for the analysis. All statistical/mathematical analyses were performed using STATISTICA (Version 12.0; StatSoft Inc. USA) and MATLAB (Version 7.7; MathWorks Inc. USA) software programs.

RSM was employed to explore the non-linear relationships between the independent (medium components) and the dependent (enzyme activity) variables. The graphs were plotted by varying the concentrations of two factors and keeping the concentrations of others factors constant (at zero level). For the dependent variable, the second-order polynomial model was obtained and expressed as Eq. 6:

6 $$\begin{array}{c}Y={{\rm{b}}}_{{\rm{0}}}+{{\rm{b}}}_{{\rm{1}}}{\rm{Xi}}+{{\rm{b}}}_{{\rm{2}}}{{\rm{X}}}_{{\rm{ii}}}+{{\rm{b}}}_{{\rm{3}}}{{\rm{X}}}_{{\rm{iii}}}+{{\rm{b}}}_{{\rm{4}}}{{\rm{X}}}_{{\rm{iv}}}+{{\rm{b}}}_{{\rm{5}}}{{{\rm{X}}}^{{\rm{2}}}}_{{\rm{i}}}+{{\rm{b}}}_{{\rm{6}}}{{{\rm{X}}}^{{\rm{2}}}}_{{\rm{ii}}}+{{\rm{b}}}_{{\rm{7}}}{{{\rm{X}}}^{{\rm{2}}}}_{{\rm{iii}}}\\ \,\,\,+\,{{\rm{b}}}_{{\rm{8}}}{{{\rm{X}}}^{{\rm{2}}}}_{{\rm{iv}}}+{{\rm{b}}}_{{\rm{9}}}{\rm{Xi}}\cdot {\rm{Xii}}+{{\rm{b}}}_{{\rm{10}}}{{\rm{X}}}_{{\rm{i}}}\cdot {{\rm{X}}}_{{\rm{iii}}}+{{\rm{b}}}_{{\rm{11}}}{{\rm{X}}}_{{\rm{i}}}\cdot {{\rm{X}}}_{{\rm{iv}}}+{{\rm{b}}}_{{\rm{12}}}{{\rm{X}}}_{{\rm{ii}}}\cdot {{\rm{X}}}_{{\rm{iii}}}\\ \,\,\,+\,{{\rm{b}}}_{{\rm{13}}}{{\rm{X}}}_{{\rm{ii}}}\cdot {{\rm{X}}}_{{\rm{iv}}}+{{\rm{b}}}_{{\rm{14}}}{{\rm{X}}}_{{\rm{iii}}}\cdot {{\rm{X}}}_{{\rm{iv}}}\end{array}$$

where, Y = response/product yield (enzyme activity), b = regression coefficient, X_i-iv_ = linear variable, X^2^ = quadratic variables, and X·X = term for interaction of the variables. The surface plots were plotted by varying the concentrations of two factors and keeping the concentrations of others factors constant at zero level. The second order polynomial model developed by RSM contained linear, quadratic and interaction terms (Eq. 6), and it was further optimized by GA. This model was saved as a function in MATLAB M-file for further optimization by using ‘ga’ function of MATLAB^50^. The input parameters of ‘ga’ function were as follows- Population Type: ‘double Vector’; Pop Init Range: [2 × 1 double]; population Size: 200; elite count: 2; crossover fraction: 1; migration direction: ‘forward’; migration interval: 20; migration fraction: 0.2000; generations: 100; time limit: Inf; fitness limit: -Inf; stall gen limit: 50; stall time limit: 20; initial population: []; initial scores: []; plot interval:1; creation fcn: @gacreationuniform; fitness scaling fcn: @fitscalingrank; selection fcn: @selectionstochunif; Crossover Fcn: @crossoverscattered; mutation fcn: {[1 × 1 function_handle] [1] [1]}; hybrid fcn: []; display: ‘off’; plot fcns: {[1 × 1 function_handle] [1 × 1 function_handle]}; output fcns: []; vectorized: ‘off’.

### Purification of COD

#### Precipitation of crude enzyme solution

The culture filtrate was precipitated at 4 °C overnight with ammonium sulfate (70% saturation). The precipitated proteins were recovered by centrifugation at 11086 g for 20 min and dissolved in 100 mM potassium phosphate buffer (pH 7.0), and finally dialyzed twice against the same buffer and concentrated with 50% sucrose solution for 3 h. All the purification steps were carried out at 4 °C temperature.

#### Ion exchange chromatography

The dialyzed sample was loaded on DEAE cellulose (Sigma, USA) column (1.3 × 16 cm), washed and pre-equilibrated with 100 mM potassium phosphate buffer (pH 7.0). The enzyme was eluted step-wise gradient of NaCl (0.1–0.5 M) in 100 mM potassium phosphate buffer (pH 7.0) at a flow rate of 0.5 ml/min. Different fractions (3 ml) were collected and estimated for enzyme activity. Positive fractions were pooled, concentrated and further purified by gel filtration chromatography.

#### Gel filtration chromatography

The pooled fractions were loaded on a sephadex G-100 column (1.8 × 50 cm) that was equilibrated and eluted with 100 mM potassium phosphate buffer (pH 7.0), 30 mM NaCl at a flow rate of 0.5 ml/min. The fractions were collected and assayed for COD activity. Active fractions were pooled, lyophilized and used as purified enzyme.

#### Polyacrylamide gel electrophoresis

Molecular weight of the purified enzyme was determined by SDS-PAGE performed at 25 mA in a Mini Protean cell (Bio-Rad Laboratories) at 25 °C using 12% polyacrylamide gel by following the method of Laemmli (1970)^51^.

### Characterization of the purified COD

The optimum pH for the stability of the purified COD enzyme was determined by incubating the enzyme (100 μg/ml) in potassium phosphate buffer (100 mM, pH 7.0) and sodium potassium phosphate buffer (10 mM, pH 7.0), respectively with different pH (4–12) range at 4 °C for 48 hr^10^. The pH of the buffer was maintained by using NaOH (1 N) and HCl (1 N). The enzyme activity without incubation was taken as 100%. The thermal stability of COD was examined by incubating the enzyme (100 μg) in 100 mM phosphate buffer (pH 7.0) at temperature ranging from (20–60 °C) for 30 min. The effect of the metal ions was measured at 50 °C in the presence of various metal ions (10 mM) concentration (Ba^2+^, Cu^2+^, Zn^2+^, Ca^2+^, Hg^2+^, Fe^2+^, Mg^2+^, Ni^2+^, Pb^+2^, Zn^+2^, Ag^2+^ Co^2+^ and Mn^2+^). The effect of organic solvents and detergents like methanol, ethanol, ethyl acetate, isopropanol, chloroform, benzene and Trion X-100, tween-80, SDS, β-mercaptoethanol, and DTT were determined by incubating 500 μl enzyme solution (100 μg/ml protein) with 500 μl of different organic solvents/detergents at 50 °C for 30 min, and residual activities were measured.

### Substrate specificity

Substrate specificity was investigated by incubating the purified enzyme with different substrates such as β-cholestanol, β-sitosterol, β-stigmasterol, and pregnenolone at the concentration of 0.5 mM in 100 mM phosphate buffer (pH 7.0) using standard assay method. The relative activity was calculated using cholesterol as a control.

### Determination of K_m_ and V_max_ values

The values of K_m_ and V_max_ were determined by Lineweaver-Burk’s plot that plots the reciprocal of velocity (1/v_0_) against the reciprocal of substrate concentration (1/[S]) containing cholesterol concentration in the range of 0.02–0.1 mM.

### UV absorption spectra

The absorption spectrum of the purified COD was taken in 100 mM potassium phosphate buffer (pH 7.0) on UV/VIS spectrophotometer (LABINDIA, UV3000).

## Electronic supplementary material

Supplementary Information

MS data file
