# Supplementary material for: Response Surface Methodology-Genetic Algorithm Based Medium Optimization, Purification, and Characterization of Cholesterol Oxidase from Streptomyces rimosus
Source: Sci Rep. 2018 Jul 19;8:10913. doi: 10.1038/s41598-018-29241-9 (PMC6053457; doi:10.1038/s41598-018-29241-9)
Supplement: Supplementary file 1 — Supplementary Information [file 41598_2018_29241_MOESM1_ESM.pdf]

SUPPLEMENTARY INFORMATION

Response Surface Methodology-Genetic Algorithm Based Medium Optimization, Purification, and Characterization of Cholesterol Oxidase from *Streptomyces rimosus*

Akanksha Srivastava[1,2], Vineeta Singh[3], Shafiul Haque[4], Smriti Pandey[1], Manisha Mishra[5], Arshad Jawed[4], P.K. Shukla[1], P.K. Singh[5], CKM Tripathi[1,6*]

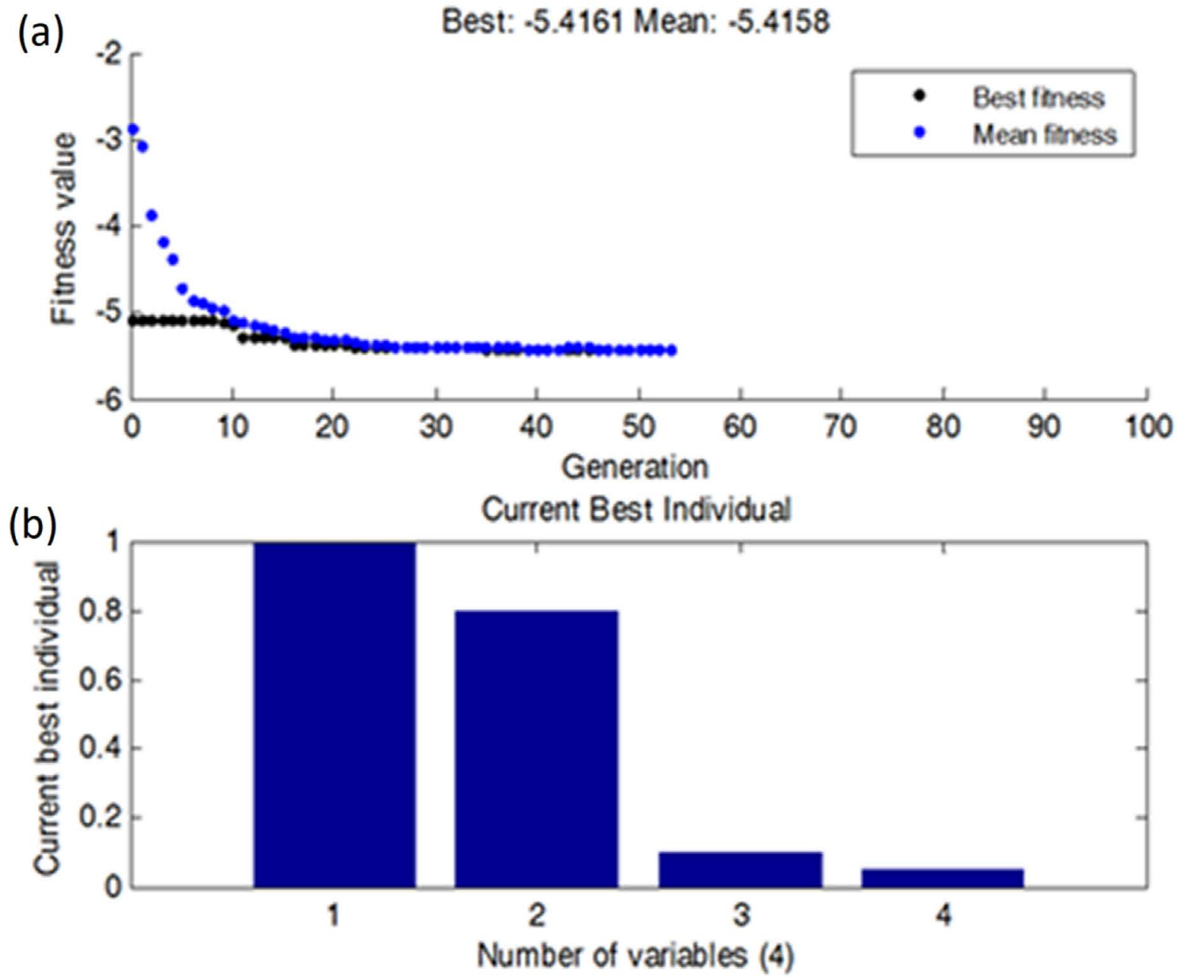

Supplementary Figure S1. Genetic algorithm showing generations till the optimum results obtained, the optimum concentration of the medium components:

(a) Current generation vs. fitness value.

(b) Current best individual vs. number of variables.

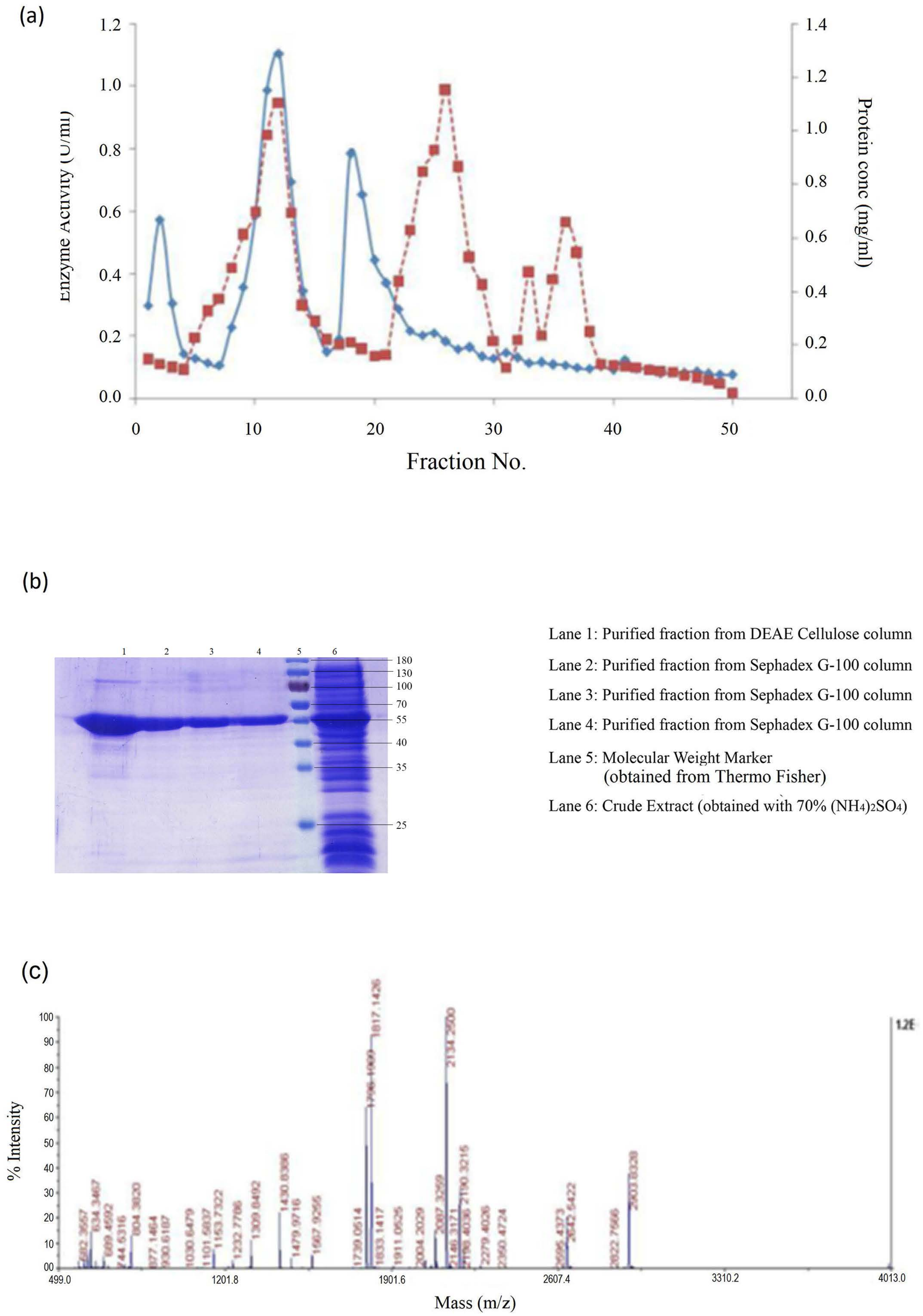

Supplementary Figure S2. (a). Elution profile of COD using DEAE-cellulose column chromatography

(b). SDS-PAGE of purified fractions and crude extract.

(c). Mass Spectroscopy (MALDI-TOF) of purified cholesterol oxidase.

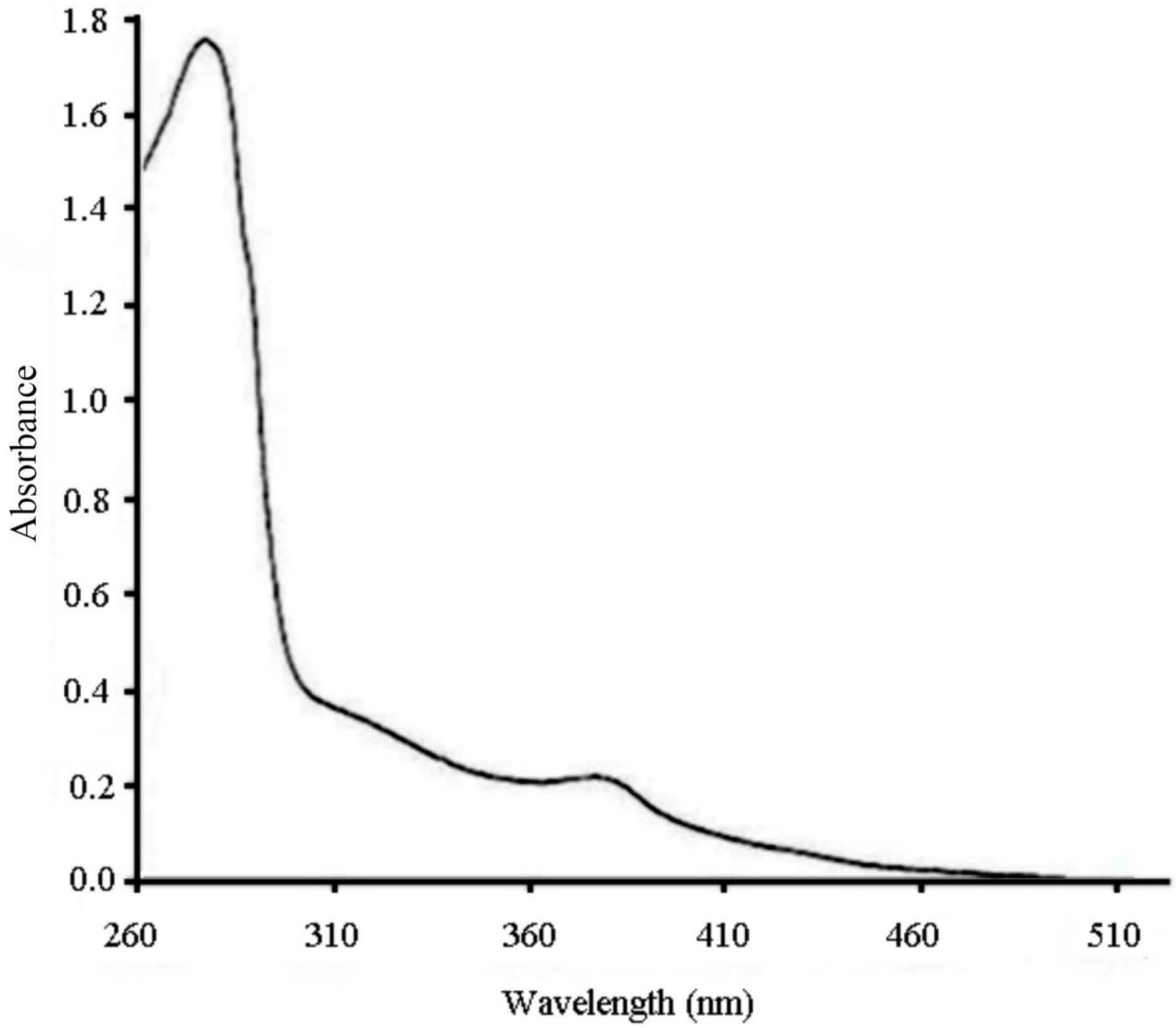

Supplementary Figure S3. UV-VIS absorption spectra with absorption maxima at 280 nm and 380 nm
