# Supplementary material for: Response Surface Methodology-Genetic Algorithm Based Medium Optimization, Purification, and Characterization of Cholesterol Oxidase from Streptomyces rimosus
Source: Sci Rep. 2018 Jul 19;8:10913. doi: 10.1038/s41598-018-29241-9 (PMC6053457; doi:10.1038/s41598-018-29241-9)

**Database:**	NCBInr
**Score:**	265
**Nominal mass (M_r):**	59185
**Calculated pI:**	8.43
**Taxonomy:**	Streptomyces sp. SA-COO

**Enzyme:**	Trypsin: cuts C-term side of KR unless next residue is P.
**Fixed modifications:**	Carbamidomethyl (C)
**Variable modifications:**	Oxidation (M)

Protein sequence coverage: 16%

Matched peptides shown in ***bold red***.

```
  1 MTAQQHLSRR RMLGMAAFGA AALAGGTTIA APRAAAAAKS AADNGGYVPA
 51 VVIGTGYGAA VSALRLGEAG VQTLMLEMGQ LWNQPGPDGN IFCGMLNPDK
101 RSSWFKNRTE APLGSFLWLD VVNRNIDPYA GVLDRVNYDQ MSVYVGRGVG
151 GGSLVNGGMA VEPKRSYFEE ILPRVDSSEM YDRYFPRANS MLRVNHIDTK
201 WFEDTEWYKF ARVSREQAGK AGLGTVFVPN VYDFGYMQRE AAGEVPKSAL
251 ATEVIYGNNH GKQSLDKTYL AAALGTGKVT IQTLHQVKTI RQTKDGGYAL
301 TVEQKDTDGK LLATKEISCR YLFLGAGSLG STELLVRARD TGTLPNLNSE
351 VGAGWGPNGN IMTARANHMW NPTGAHQSSI PALGIDAWDN SDSSVFAEIA
401 PMPAGLETWV SLYLAITKNP QRGTFVYDAA TDRAKLNWTR DQNAPAVNAA
451 KALFDRINKA NGTIYRYDLF GTQLKAFADD FCYHPLGGCV LGKATDDYGR
501 VAGYKNLYVT DGSLIPGSVG VNPFVTITAL AERNVERIIK QDVTAS
```

Unformatted sequence string: 546 residues (for pasting into other applications).

Residue Number   Increasing Mass   Decreasing Mass

Query	Start – End	Observed	Mr(expt)	Mr(calc)	Delta	M	Score	Expect	Rank	U	Peptide
46	107 – 124	2087.3267	2086.3194	2086.0960	0.2235	1	17	1.3e+02	3		K.NRTEAPLGSFLWLDVVNR.N
37	109 – 124	1817.1423	1816.1350	1815.9519	0.1831	0	48	0.11	1		R.TEAPLGSFLWLDVVNR.N
50	221 – 239	2134.2505	2133.2432	2133.0354	0.2079	0	119	9.3e-09	1		K.AGLGTVFVPNVYDFGYMQR.E

Query	Start – End	Observed	Mr(expt)	Mr(calc)	Delta	M	Score	Expect	Rank	U	Peptide
60	340 – 365	2642.5430	2641.5357	2641.2555	0.2803	0	33	2.5	1		R.DTGTLPNLNSEVGAGWGPNGNIMTAR.A
64	506 – 533	2903.8333	2902.8260	2902.5440	0.2820	0	64	0.0015	1	U	K.NLYVTDGSLIPGSVGVNPFVTITALAER.N

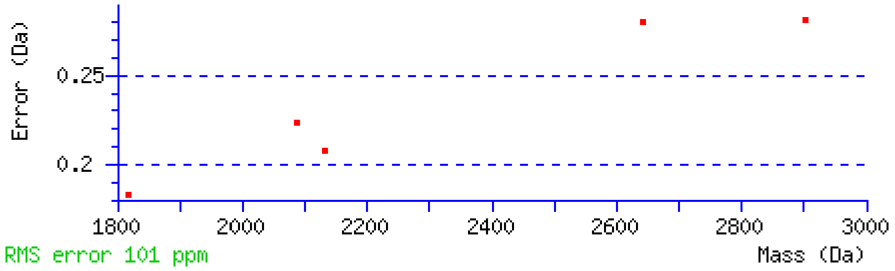

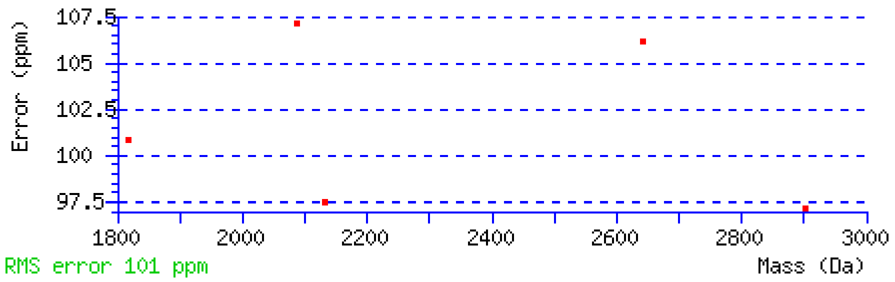

Supplement: Supplementary file 2 — MS data file [file 41598_2018_29241_MOESM2_ESM.pdf]
